# Tracing Children with Blindness and Visual Impairment Using the Key Informant Survey in a District of North-Western Nigeria

**DOI:** 10.4103/0974-9233.71601

**Published:** 2010

**Authors:** Nasiru Muhammad, Nuhu M. Maishanu, Aliyu M. Jabo, Mansur M. Rabiu

**Affiliations:** Ophthalmology Department, Sir Yahaya Memorial Hospital, Birnin Kebbi, Nigeria; 1 Specialist Hospital, Sokoto, Nigeria; 2 Sokoto State Eye Care Programme, Sokoto, Nigeria; 3 National Eye Centre, Kaduna, Nigeria

**Keywords:** Childhood Blindness, Eyecare Program, Inclusive Education, Key Informant Survey, Nigeria, Prevention of Blindness Program

## Abstract

**Purpose:**

To identify children with irreversible blindness in a district of northern Nigeria for enrolment into an inclusive education pilot project.

**Materials and Methods:**

Using key informants (KIs) working and residing within the communities, children with blindness and visual impairment in Gwadabawa local government area (LGA) were identified and then examined by a team of ophthalmologists/optometrists. Data analysis was performed manually using simple percentages and proportions.

**Results:**

Sixty children were reported with visual problems by parents/guardians of whom 58 (97%) were examined. Twenty children (35%) were blind, 17 (29%) were irreversibly blind, and 9 (16%) had low vision (<6/18 to 3/60) with presenting vision. The major causes of childhood blindness were corneal opacity/phthisis bulbi (75%), and cataract (15%). The cause of irreversible blindness in these children was largely preventable (80%) as it was due to childhood-related illnesses, such as vitamin A deficiency and measles.

**Conclusions:**

The major causes of childhood blindness in the study area were avoidable and the use of KI survey in this study provided an opportunity for service delivery.

## INTRODUCTION

Sokoto state located in northern Nigeria, in collaboration with Sight Savers International is implementing comprehensive eyecare program services since 2005 based on the principles of VISION 2020: the Right to Sight.^1^ The program is providing community-based eyecare services, especially in the rural areas of the state and is planning to introduce inclusive education for the irreversibly blind children.^2^ A survey was conducted to identify children with blindness/visual impairment using the key informants (KIs) method. This enabled treatment of those with treatable blindness/low vision and referral of those with irreversible blindness for inclusive education.

The pilot district has a population of 246,402, 50% of which are children in the age group of 0–15 years.^3^ An Ophthalmic Nurse (ON) renders secondary eyecare services in the district and 10 community health extension workers (CHEWs) provide primary eyecare services as trained integrated eyecare workers (IECWs). This article reports the use of KIs to trace blind children in Gwadabawa Local Government Area (LGA), a district of Sokoto State, Nigeria. KI surveys have been described as an alternative to population-based studies in evaluating community-based programs, and needs assessments.^4^ This study aimed to identify children with blindness and visual impairment in Gwadabawa LGA of Sokoto State, Nigeria. The specific objectives were as follows: (i) to find children with treatable blindness and visual impairment and refer them for appropriate treatment; (ii) to find children with irreversible blindness for enrolment in an inclusive education program in the LGA; and (iii) to determine the causes of childhood blindness in the LGA.

## MATERIALS AND METHODS

Using a community mobilizer (CM) and KIs, a cross-sectional survey was conducted to trace children with visual impairment and blindness in Gwadabawa LGA of Sokoto State in October-November 2008. The ON working in the LGA was nominated as the CM for the survey because she lives and works in the LGA. The CHEWs working in the health care centers across the LGA were used as KIs as they cover all the primary health care centers across the LGA. It was expected that the CM would mobilize the KIs to search for these children as she has been working with the KIs in eyecare activities.

### Survey organization

Over a four-week period, the following steps^4^ were used to find blind/visually impaired children in the LGA: mapping and community sensitization; training of KIs; and case finding and health education. Eye examination was then conducted at an arranged date and venue. The LGA is administratively divided into 3 districts consisting of 11 wards.

### Mapping and community sensitization

Mapping involved selecting and distributing the KIs to cover the wards. Local authorities, including the district heads, were informed and sensitized on the survey and their support and participation was solicited. They were requested by the team, to convey the information to their subjects. This was followed by sensitization of identified groups in the communities, which included ward heads, political leaders, traditional/religious leaders, other health workers in the LGA, and teachers (Islamic and conventional schools). They were sensitized on the potential benefits of finding children with visual problems to both the society and their families.

### Training of key informants

After selection of the 22 KIs (2 per ward), a date and venue were fixed for their training. The one-day training was conducted by the ophthalmologist supported by the CM. The KIs were assigned to wards based on work station. The content of the training included basic knowledge on common causes of childhood blindness and benefits of treatment (surgical, medical, or optical), benefits of inclusive education and access to other services, and visual acuity (VA) assessment. The vision assessment in pre-primary school children (0–5 years) was determined mainly by history from the parents. Children in the age group of 6–15 years underwent VA testing with the Snellen E-chart. KIs were also trained on the procedure for filling a roster for identified children, and some skills on health education, communication, and team work. A post-training assessment was conducted to ensure that the KIs were all proficient in filling the roster for the identified children and the technique of health education and communication. After the training, a date and venue were fixed for the eye examination by the ophthalmic team at the LGA headquarters.

### Subject identification and health communication

Over a 10-day period, town criers (usually working for the ward head) and the KIs spread the information in the villages that a search for children with visual impairment/blindness was ongoing in the communities and solicited the cooperation and support of parents. Members of the communities and parents were also solicited to bring any child who they believed to have visual problems to the health care center anytime or to the village head on a specified day. The KIs registered all the children brought to the village head/health care centers and offered health education to the parents/guardians. They recorded the names, age, sex, and full address of all the children and their guardians.

Both the ophthalmologist and community mobiliser were on supervisory and monitoring visits to all the wards to discuss progress and problems with the activities of the KIs. They also selected some households on the list to visit to ascertain and verify the correct address.

KIs also informed parents of the day and venue chosen for eye examination by the ophthalmic team. Parents were also assured that they will be reimbursed for expenses they incurred for transport on the day of the examination.

### Eye examination

The eye examination was conducted at the Eye Clinic of the Primary Health Center in the LGA capital. The team comprised Ophthalmologist, optometrist, and three ONs. The eye examination was conducted in stages.

#### Registration and visual acuity assessment

These were done by the ONs. The name, age, sex, and contact address of each child were recorded into the data collection form, that is, the World Health Organization prevention of blindness (WHO/PBL) record.^5^ For verbal children (4–15 years), presenting vision was assessed with the Snellen E-chart after explanation and demonstration to the children. The chart was placed at a distance of 6 m and each eye was tested separately, while care was taken to ensure that the other eye was completely covered. None of the children had a history of using spectacles. All findings were recorded in the data collection form. Correct identification of at least 3 optotypes on consecutive presentations was considered as having read a line. Eyes that failed counting fingers at 1 m were tested for hand motion and then perception of light where necessary. Any eye that failed a VA of 6/18 was tested with a pinhole by the optometrist and then refracted if there was an improvement with the pinhole. Manual retinoscopy was performed followed by subjective refraction.

For preverbal children (0–3 years), the vision was tested by assessing the child’s ability to fixate a light source and blindness in such children was defined as the inability to fixate a light source.

The operational definitions were based on the WHO/PBL coding instructions for eye examination record^6^ except for definition of blindness and low vision, which was defined based on recent recommendations^7^ to reflect real-life situations. Blindness was defined as a presenting VA of less than 3/60 (CF at 3 m) in the better eye, whereas low vision was defined as a presenting VA of less than 6/18 to 3/60.^7^ A basic eye examination was first performed by the ophthalmologist and then an evidence of previous eye surgery or features of xerophthalmia were assessed. The examination was undertaken with a penlight, direct ophthalmoscope, and a magnifying loupe.

The cause of low vision or blindness was then determined for each eye and then the principal cause for the child was assessed. If the pathologies responsible for visual impairment in both the eyes were different, the principal cause was that which is most easily curable, if not then the most preventable. The necessary action required for each eye was also determined. A cause of blindness and low vision was determined for both etiologic (historical) and anatomic groupings using the World Health Organization (WHO) survey definitions.^6^

All the findings were appropriately recorded into the WHO/PBL eye examination record.^5^

Detailed contact address of the blind children and their guardians were recorded and appropriate referral was made for low vision assessment, cataract surgery, and further evaluation at the base hospital. Arrangements were made to issue spectacles to those children identified with refractive errors. Those identified to be irreversibly blind were enlisted for inclusion in the inclusive education program. Chloramphenicol eye drops and tetracycline eye ointments were issued to children where appropriate. Children who failed to come for the scheduled eye examination were contacted at their homes and examined. The data were analyzed manually using percentages and proportions.

#### Ethical considerations

Permission to conduct the survey was obtained from the State Ministry of Health and the local authorities. Consent was given by the parents/guardians of the children before examination.

## RESULTS

### Demographic characteristics of the children

Sixty children were registered by the KIs as having vision problems of whom 58 (97%) were examined. Forty-one children (68%) were brought for examination as scheduled, whereas 17 (28%) were located and examined at home. One died before the examination day, and another one was said to be blind in one eye and had gone to a nearby market. Boys constituted 62% of the children as shown in Table 1.

**Table 1:** Age–sex distribution of examined children

Age group (years)	Boysn (%)	Girls n (%)	Total n (%)
0-5	7 (12)	2 (3.4)	9 (15.5)
6-10	8 (13.8)	7 (12)	15 (25.9)
11-15	21 (36.2)	13 (22.4)	34 (58.6)
Total	36 (62)	22 (37.9)	58 (100

### Presenting visual acuity among the children

A total of 20 (34%) children were blind, whereas 9 (15.5%) children had low vision [Table 2]. This gives a prevalence of approximately 0.02% for childhood blindness in the study area.

**Table 2:** Presenting visual acuity in the examined children

Vision category	Number (%)
Blind	20 (34)
Unilateral blindness	9 (15.5)
Low vision	8 (14)
Unilateral low vision	12 (21)
Normal	9 (15.5)
Total	58 (100)

### Causes of blindness and low vision

The major cause of blindness was corneal opacity (55%), whereas that for low vision was refractive error and corneal opacity each accounting for 44% [Table 3]. Those blind from phthisis bulbi and anterior uveitis were absolutely blind (NPL), whereas the rest had dense scarring of the entire cornea but perceived light on vision testing in the better eye.

**Table 3:** Causes of blindness and low vision

Cause	Blindness (%)	Low vision (%)
Corneal opacity	11 (55)	4 (44.4)
Cataract	3 (15)	1 (11.1)
Phthisis bulbi	4 (20)	0
Anterior uveitis	2 (10)	0
Refractive error	0	4 (44.4)
Total	20 (100)	9 (100)

### Etiology and anatomic site of abnormality

Table 4 shows the anatomic site of abnormality and the underlying etiologic causes of blindness and low vision among the study sample. The most common anatomic site of a blinding abnormality was the cornea (55%), whereas the most common etiology was a childhood factor (75%), which included measles, vitamin A deficiency, infectious keratitis, and trauma.

**Table 4:** Anatomic site of abnormality, underlying etiology, cause of blindness, and low vision

	Blindness (%)	Low vision (%)
Anatomic site		
Whole globe	4 (20)	0
Cornea	11 (55)	4 (44.4)
Lens	3 (15)	1 (11.1)
Uvea	2 (10)	0
Refractive	0	4 (44.4)
Etiology		
Hereditary	2 (10)	4 (44.4)
Childhood factor	15 (75)	5 (55.6)
Unknown (uveitis)	2 (10)	0
Couching	1 (5)	0
Cause		
Preventable	16 (80)	4 (44.4)
Treatable	4 (20)	5 (55.6)
Unavoidable	0	0

The causes of blindness were mostly preventable (80%), whereas the causes of low vision were mostly treatable (55.6%). Table 5 shows the action required for the examined children.

**Table 5:** Current action needed in the children with visual impairment/blindness

Vision category	Aetiology (n)	Action needed
Blind	Cataract (3) Corneal opacity/phthisis (17)	Cataract surgery Inclusive education
	Corneal opacity/phthisis (17)	Inclusive education
Low vision	Cataract (1)	Cataract surgery
	Corneal opacity (4)	Further evaluation
	Refractive error (4)	Spectacles
Unilateral blindness	Cataract (1)	Cataract surgery
	Corneal opacity (7)	Further evaluation
Unilateral low vision	Corneal opacity (12)	Further evaluation

## DISCUSSION

Children who are blind need to be identified as early as possible so that they can be examined and treated, referred, or rehabilitated. This is crucial if they are to have the best possible chance of proper childhood development, education, and participation in broader social life.^4^ The use of the KI method in this study was impressive as children with blindness and visual impairment were traced during the survey.

Of the 58 children who were examined, 17 (29%) were recommended for enrolment in inclusive education as they were irreversibly blind from phthisis bulbi, total corneal opacity, or complicated uveitis. The cause of irreversible blindness in these children is largely preventable (80%) as it was due to childhood-related illnesses, such as vitamin A deficiency and measles. The immunization coverage for measles in the study area has been low over the last few years as the records show coverage as low as 7% in 2000 to only 58% in 2005.^8^ A low coverage was attributed to rejection of vaccines by many parents due to suspicion of its potency and benefit. There had been annual outbreaks of measles epidemics in many communities over the years. It is therefore not surprising that the major causes of irreversible blindness are corneal opacities and phthisis bulbi. This finding is comparable to the WHO’s estimate of corneal blindness accounting for 70% of childhood blindness in Africa.^9^ It, however, contrasts to the findings in Bangladesh where there were more treatable causes (45%) than preventable causes (26%).^10^

Similar to our findings (55%), a study at an eye hospital in Nigeria also reported corneal blindness in 54.8%^11^ of blind children seen at the hospital. However, our findings differ from the observations that 21.4% of children in schools for the blind had corneal blindness in southeastern Nigeria.^12^ This may be due to our selection of locale for our study (northwestern Nigeria), whereas the schools for the blind are in the southeast where literacy level is higher than in the northwest region of the country and the national health indices are generally worse in the northwest. Additionally, the reported proportion of cataract blindness in our study (15%) is similar to that reported in the eye hospital study (12.9%)^11^ but lower than the findings in the schools for the blind (23.5%).^12^

All the blind children in this study had preventable blindness, in contrast to the reports from other institution-based studies in Nigeria where 58.6%^11^ and 74.5%^12^ were from avoidable causes. This may be a result of the population-based nature of our study and the likely socioeconomic factors that affect the health-seeking attitude and the mortality rate of children in remote communities.

An additional 8 (14%) children were found to be unilaterally blind, also from preventable causes. This underscores the importance of strengthening primary health care by enhancing primary eye care, antenatal care, immunization, and vitamin A supplementation to prevent the occurrence of avoidable blindness.

Of those with low vision, 45% were refracted and provided with spectacles, whereas those with corneal opacities and vision better than light perception were referred for further evaluation at the base hospital. Identification of these children in these communities provided treatment for those with treatable causes that included cataract surgery and spectacles (service delivery) and inclusive education for those irreversibly blind. It has been observed that in most developing countries only 10% of blind children^13^ are in special schools, thus the alternative scenario would have led these children into begging as is the common tradition in these communities where blind children are used as a source of income through begging, becoming a social liability to themselves, their families, and the general society.

It is therefore recommended that similar surveys be conducted in the remaining LGAs in the state to identify children needing ophthalmic services and/or education. The local authorities and their partners need to strengthen primary health care activities and also intensify efforts to ensure wider immunization coverage in the communities through public education.

The major causes of childhood blindness in the study area are largely avoidable from childhood-related illnesses and the use of KI survey in this study provided an opportunity for service delivery to the hard-to-reach less privileged children.
